# Advance Care Planning in Dialysis Patients With a Conversation Game

**DOI:** 10.1016/j.ekir.2025.11.001

**Published:** 2025-11-10

**Authors:** Anne Dufey Teso, Gora Da Rocha Rodrigues, Catherine Bollondi Pauly, Pascale Lefuel, Monica Escher, Christine Clavien

**Affiliations:** 1 Division of Nephrology and Hypertension, Department of Medicine, Geneva University Hospitals, Geneva, Switzerland; 2 HESAV School of Health Sciences - Vaud, HES-SO University of Applied Sciences and Arts Western Switzerland, Lausanne, Switzerland; 3 Division of Professional Practices, Care Directorate, Geneva University Hospitals, Geneva, Switzerland; 4 Division of Palliative Medicine, Department of Rehabilitation and Geriatrics, Geneva University Hospitals; Unit for Development and Research in Medical Education (UDREM), Faculty of Medicine, University of Geneva, Geneva, Switzerland; 5 Institute for Ethics History and the Humanities, Faculty of Medecine, University of Geneva, Geneva, Switzerland

**Keywords:** advance care planning, communication, conversation game, documentation, patients’ values, shared decision-making

## Abstract

**Introduction:**

To support advance care planning (ACP), a conversation game, *Anticip'action*, and a connected 3-step ACP intervention using the game were developed. This study assessed the acceptability, feasibility, and effect of the intervention with patients undergoing dialysis at a tertiary hospital.

**Methods:**

Dialysis nurses received a 10-hour training in ACP and use of the card game *Anticip’Action.* All patients on dialysis in the nephrology division were eligible. Acceptability and feasibility were assessed using questionnaires and note taking. Effect was assessed using pre-post number of advance directives (ADs), number of patients designating a surrogate, quality of written ACP documentation, patients’ responses to the 9-items ACP Engagement Survey, and patients’ assessment of their ACP documentation.

**Results:**

Twelve nurses conducted ACP interventions. Thirty-three patients (87% of eligible patients) accepted to be included, 25 (75%) started the ACP intervention, 23 completed it, and 18 answered all questionnaires. 5 withdrawals were due to premature death. Nurses and patients rated the training, the intervention and the card game as good and highly acceptable: mean scores ranged from 2.87 to 4.17 on a 5-point Likert scale questions. Organizational difficulties were reported by nurses. Significant increases were observed on uploaded ADs (+ 30%), designated health care surrogates (+ 80%), patients reporting end-of-life planning actions (12/19), patients’ engagement in ACP (+ 1.04 on 1–5 scale, *P* ≤ 0.001), quality of ACP documentation (+ 2.68 on a 1–5 scale, *P* ≤ 0.001), and patients’ evaluation of their ACP documentation (3.44 ± 1.04 on 1–5 scale).

**Conclusion:**

The 3-step ACP intervention using *Anticip’action* showed good implementation potential in clinical context.

## Graphical abstract

**Figure:**
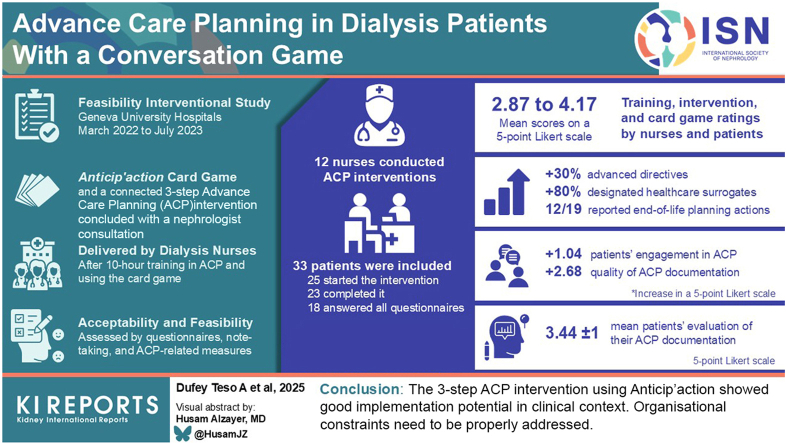

Dialysis can prolong the life of patients suffering from end-stage renal disease, with a 5-year survival probability of 50% to 57% in Switzerland.^1^ However, this renal replacement therapy is burdensome and associated with significant morbidity and mortality, compared with the general population. For example, 20- to 24-year-old patients on dialysis have a 40-year shorter remaining life expectancy compared with the general population of the same age.^2^ This increased risk of premature death is only partly reduced by kidney transplantation.

These data indicate that patients on dialysis can benefit from being engaged in patient-centered ACP programs. ACP is promoted in professional, national, and international recommendations for the management of patients with chronic diseases.^3^^,^^4^ It is a process that “allows people to define their goals and preferences for future medical treatment and care, discuss them with their families and health care providers, and record and adapt them when appropriate.”^5^

ACP can empower patients to express what matters to them, improve communication with family members and health care professionals, and increase completion rate of ADs.^6^ In case of impaired decision-making capacity or sudden deterioration of health, it helps families and physicians to make decisions that are aligned with patients’ values.^7^ A recent ACP intervention conducted in dialysis scenters significantly improved patients’ preparedness for end-of-life decision-making, reduced decisional conflict, and enhanced surrogate decision-makers’ confidence.^8^^,^^9^ In another study, patients and their families insisted that ACP should be carried out early, before any therapeutic decision was taken, and should continue throughout the course of the disease.^10^ However, ACP and systematized end-of-life discussions are seldomly performed, because of numerous conceptual, emotional, and institutional barriers.^11^, ^12^, ^13^ For example, a qualitative study involving health professionals and aged patients with advanced chronic kidney disease^14^ found that unclear concepts lead health professionals to view ACP as mere documentation, leaving patients to discuss their values and goals of care outside the medical context. Patients were often overly overconfident that their preferences were understood by their nephrologists and relatives. Further, barriers included professionals’ doubts of the relevance of ACP, organizational difficulties (lack of time or dedicated space), or insufficient training to help professionals address the issue effectively and alleviate their anxiety.^14^, ^15^, ^16^ Overall, there is a need for more institutional support and for the development of policies and procedures that can be systematically deployed and routinized in care contexts.^4^^,^^11^^,^^16^

Taking inspiration from studies indicating that serious games are helpful tools to support ACP conversations,^17^^,^^18^ an interprofessional research team (based at Geneva’s Institute of Ethics, History, and Humanities and at the Nursing Directorate of Geneva University Hospitals) developed the discussion tool *Anticip'action*^19^ using iterative rounds of development and critical feedback from both experts and patients. *Anticip'action* is a card game, inspired by the *Go Wish*,^20^ which contains additional rules and contents related to values and goals of care. It is designed for use in a wide range of settings: in clinical contexts with the support of health professionals, but also independently at home with loved ones. The game helps users identify their values and priorities, clarify their goals of care, and develop concrete action plans. This conversation tool aims to facilitate the initiation of ACP and end-of-life discussions and to increase patients’ empowerment during these conversations. A short description of the game rules is provided in Figure 1. Previous studies have shown that the game is easy to use^21^ and well-received in community settings.^22^

**Figure 1:**
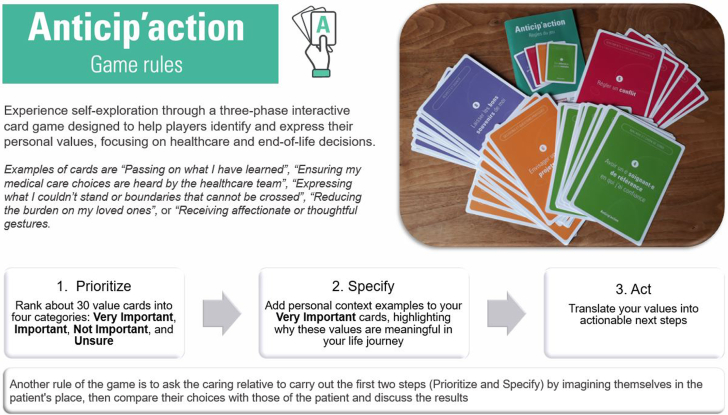
Short description of the game rules of *Anticip’action*.

In parallel, a 3-step ACP intervention adapted to patients on dialysis was initiated and pilot-tested with the similar game *Go Wish*.^20^ The intervention was adapted to patients on dialysis, included the use of a conversation game, and the instruction to document discussions in the dedicated ACP section in the medical record.^23^ Patients on dialysis are an ideal population for a structured ACP intervention because of their regular presence in care facilities, enabling a multistage process without additional travel or organizational burdens.

The aim of this study was to assess the feasibility, acceptability, and effect of the 3-step ACP intervention with the use of the conversation tool, *Anticip’action*, taking place in a nephrology unit and involving patients, relatives, nurses, and nephrologists.

## Methods

### Ethics Approval and Consent to Participate

The study was approved by the ethics committee of the Geneva Commission Cantonale d’Ethique de la Recherche (Req-ID 2021-02078: 21.02.2022). This study was conducted in accordance with the Declaration of Helsinki. Willing participants were informed of the study aims and procedures and signed an informed consent form. Patients’ responses to questionnaires were recorded and stored in a secure data collection program that complies with clinical studies requirements. Their responses were handled with utmost discretion and only accessible to authorized personnel. Data were coded and the code key destroyed at the end of the data analysis phase. No identifying information is kept in the remaining work files.

### Recruitment and Study Procedure

The study took place in the nephrology unit, at Geneva University Hospitals and involved 2 types of participants: patients, and health professionals (Table 1).

**Table 1:** Inclusion and exclusion criteria

Inclusion criteria	Patient	Health professional
- has provided free and informed written consent	X	X
- aged ≥ 18 yrs	X	X
- fluent understanding of French (= language of the game used)	X	X
- is diagnosed with end-stage renal disease	X	
- is undergoing ambulatory hemodialysis or peritoneal dialysis in the nephrology unit	X	
- has the decision-making capacity regarding ACP	X	
- works in the study site (nephrology unit, HUG)		X
- has followed the training sessions planned in the study protocol		X
In addition, for interviews and focus group: has been involved in the ACP intervention and followed ≥ 2 meetings	X	X
For “external observer” focus group only: is a health professional working in the nephrology unit		X
Exclusion criteria		
- Has lost the capacity to make decisions about advance care planning	X	

ACP, advance care planning; HUG, Geneva University Hospitals.

Nurses were recruited following an internal introductory talk about ACP. All interested nurses were invited to a 1-hour information session about the study and signed an informed consent. They received training, which included one 45-minute online module and 3 x 3-hour training sessions (Supplementary Material). The training aimed to help nurses to master the concepts and philosophy of ACP, to work on their approach and attitude in this process, to document ACP information in patients’ record, to master the game *Anticip’action*, and to conduct the 3-step ACP intervention described in Table 2. Following the training, according to their affinities and professional availability, nurses were assigned patients to conduct the 3-step ACP intervention. Allocated time was secured in collaboration with the heads of the nephrology unit. Five times during the study, a continuous training opportunity was offered to nurses; they could participate in a 1-hour feedback and exchange meeting with 2 experienced ACP coaches (CB and PL). These sessions provided opportunities to discuss difficulties or to share positive experiences. Personalized informal supervision sessions were provided upon demand. At the end of the study, nurses were asked to evaluate the ACP training, the intervention, and the card game in an online questionnaire.

**Table 2:** Description of the 3-step ACP intervention

Step	Participants	Duration	Location	Description
1	Patient and nurse	60 min	During a dialysis session, if possible, in an individual room (if not, in the common dialysis room)	Explanation of ACP process and rules of *Anticip’action*; playing the first step of the game (discovery of themes written on cards and selection of the maximum 10 most important to the patient); at the end of the session, if wished, the patient takes the game home to refine or complete the selection and to allow the close relative to play the game *as if it were the patient* and then compare the outcome with the patients’ selection; schedule next meeting (step 2), if possible 2 weeks later.
2	Patient, relative (if wished), and nurse	60 min	During a dialysis session, if possible, in an individual room	Discussion of the results of the game played by the patient and (if relevant) by the relative; specify patients’ values, preferences, fears, priorities related to the selected themes; discuss goals of care; schedule next meeting (step 3) as soon as possible
3	Patient, nurse, and referral doctor	20–40 min	During an ordinary consultation	Answer medical questions; finalize the ACP procedure (goals of care, medical decisions); if wished, write AD.

ACP, advance care planning; AD, advance directive.

**Figure 2:**
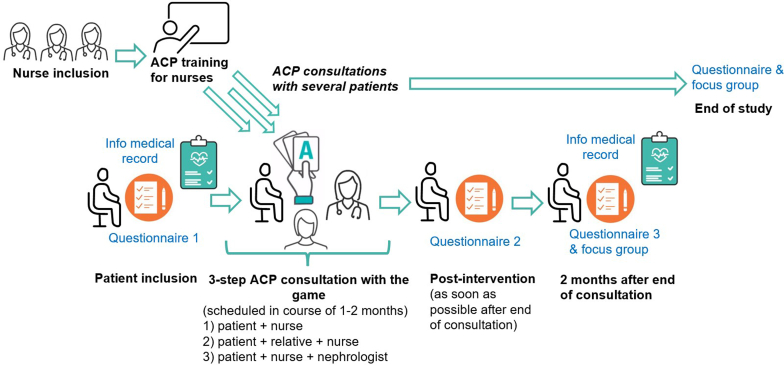
Flow diagram of the study procedure involving patients, trained nurses, and patients’ relatives. ACP, advance care planning.

Patients were approached and informed individually by a member of the research team working in the nephrology unit (PL). Upon written consent, they provided sociodemographic information and reported their baseline engagement in ACP in a questionnaire (Figure 2) before being assigned to a trained nurse to carry out the 3-step ACP intervention (Table 2). This intervention was conducted during scheduled dialysis treatment sessions. Thereafter, patients evaluated the intervention and the game in a postintervention questionnaire. Two months later, they reported their engagement in ACP in a questionnaire. Because dialysis can be exhausting, all questionnaires were completed with the help of a research assistant who could support patients’ understanding and alleviate writing burden. In addition, medical and documented ACP information was retrieved from patients’ medical record at inclusion, and 2 months after the end of the ACP intervention. The first questionnaire, and the 3-step intervention were pilot-tested with one patient to make final adjustments.

Before the study began, nephrologists likely to be involved in the third step of the ACP intervention attended a presentation of the study procedures and were introduced to the game. They did not have to adapt their usual working schedule because the third step could be planned as a regular consultation. Before that consultation, the nurse provided a summary of the patients’ values, goals of care, and questions. Based on this information, the consultation focused on clarifying patients’ treatment preferences, considering their medical situation.

### Quantitative Data Collection

Patients’ responses to questionnaires and data retrieved from medical records were collected by the research assistant and directly reported on REDCap, a secure electronic data capture program hosted at the Geneva University Hospitals that complies with clinical studies requirements.^24^^,^^25^ Nurses’ online questionnaire was also set up with REDCap.

Data included administrative information (inclusion rate, retention rate, participants’ completion, and timing of all steps of the procedure), involvement of patients’ close relatives, and sociodemographic and medical information.

Acceptability and feasibility outputs (Table 3 and Supplementary Material) were recorded using questionnaires. They included the evaluation of the quality of the ACP training for nurses (adequacy of the content, feeling supported, and reported knowledge increase), of the 3-step intervention (organizational aspects and perceived impact), and of *Anticip'action* (relevance, understandability, emotional impact, overall endorsement, and perceived impact – evaluated using 5-point Likert scales items partly taken from the *user* MARS questionnaire*,* validated for reliability and objectivity^26^).

**Table 3:** Description of acceptability and feasibility measures (individual items or scores) used to evaluate the ACP training, the 3-step ACP intervention, and the game *Anticip’action*

Name of measures (item or score)	Number of items	Description of content	Patients	Nurses
			Mean ± SD (number f responses)	
TRAINING	4	Does the training (1) contain adequate content, (2) makes you feel supported, (3) allows you to master the intervention and (4) the game?		4.08 ± 0.61 (*n* = 10)
***3-stepintervention***				
IMPACT OF INTERVENTION	3	Did the intervention help (1) clarifying values and preferences, (2) expressing them, and (3) plan related actions?	3.89 ± 0.46 (*n* = 20)	4.1 ± 0.67 (*n* = 10)
ORGANISATION	6	(1) Was it easy to find time for the ACP consultations? Was the location (2) adequate and (3) comfortable? Were the (4) number of ACP meetings, (5) their length, (6) and the time space between meetings adequate?	4.06 ± 0.4 (*n* = 20)	2.87 ± 0.49 (*n* = 10)
***Game Anticip’action***				
RELEVANCE OF GAME	2	Do you find the game (1) relevant to support an ACP procedure and (2) is the content of its cards relevant?	3.92 ± 0.67 (*n* = 20)	3.95 ± 0.37 (*n* = 10)
UNDERSTANDABILITY	2	Are (1) the rules of the game and (2) the cards easy to understand?	4.03 ± 0.55 (*n* = 20)	3.7 ± 0.71 (*n* = 10)
UPSETTING	1	Are the cards upsetting?	3.3 ± 1.15 (*n* = 20)	2.9 ± 0.74 (*n* = 10)
INTRUSIVENESS	1	Are the cards intrusive?	2.25 ± 1.1 (*n* = 20)	2.5 ± 0.85 (*n* = 10)
IMPACT OF GAME^a^	6	Does the game increase users’ (1) ACP awareness, (2) knowledge, (3) attitude,(4) help seeking, (5) intention to change, and (6) likelihood of change in ACP?	3.89 ± 0.37 (*n* = 20)	4 ± 0.56 (*n* = 10)
RECOMMENDATION^a^	3	Would you recommend the game to different categories of users: (1) patients, (2) close relatives, (3) health professionals?	3.73 ± 0.53 (*n* = 20)	3.97 ± 0.92 (*n* = 10)
STAR EVALUATION^a^	1	What is your overall (star) rating of the game?	4.17 ± 0.55 (*n* = 20)	3.8 ± 0.79 (*n* = 10)

ACP, advance care planning.

Report of participant's mean responses on individual items or scores. All items allow for answers on a 1-5 scale, with 1 = not at all and 5 = yes extremely.

a These questions were adapted from the *MARS* questionnaire.

The effect of the intervention was assessed with patients’ pre-post responses to the 9-item ACP Engagement Survey (validated questionnaire, using 5-point Likert scales items, to detect patients’ change on contemplation, self-efficacy, and readiness to name a surrogate, talk about ACP with the doctor and surrogate, and write AD),^27^^,^^28^ reported additional end-of-life planning actions, and information retrieved from patients’ medical record: pre-post number of uploaded ADs and named health care surrogates, pre-post scores in quality of written ACP documentation about the following: (i) patients’ values, (ii) end-of-life preferences, and (iii) instructions for life-sustaining treatment (evaluated independently by 1 nurse and 1 physician on a 1 to 5 scale ranging between “not at all clear” and “extremely clear” (Supplementary Methods). Data on patients’ evaluation of the adequacy of ACP documentation in their medical record were collected.

Detailed methodological information about translation of questionnaires (ACP Engagement Survey and MARS), score calculation, and statistical analysis (paired- or unpaired Welch *t* test, McNemar chi-Square) is provided in Supplementary Methods.

### Qualitative Data Collection

To obtain more qualitative information about acceptability and feasibility, the study protocol included recorded focus groups and individual interviews. Following a convenient chronological sample strategy, most patients (15/18) who completed the whole procedure were invited to an interview. At the end of the study, all nurses and patients’ referral physicians involved in the third step of the ACP intervention were invited to a focus group or individual interview, as appropriate given their organizational constraints. Moreover, all nurses who were not involved in the study but worked in the nephrology unit were invited to participate in an “external observer” focus group. Detailed method and results are reported in a separate paper (forthcoming).

In addition, the research assistant who collected questionnaire responses and the ACP coaches (PL and CB) who provided continuous supervision as part of the training to nurses were instructed to take regular notes of any relevant information related to the acceptability and feasibility of the intervention and report it to CC during supervision meetings. Notably, spontaneous comments made by patients while answering the study questionnaires were reported by the research assistant in commentary spaces in the Redcap data collection system (0–4 inputs per patient). Moreover, qualitative comments were written by nurses in a final open commentary space concluding the online questionnaire (15 inputs in total). These data were analyzed separately by 2 authors (CC and AD) with a thematic analysis procedure, then compared and discussed in case of divergence. The aim was to retrieve qualitative information to illustrate or explain the quantitative results obtained.

## Results

### Participation and Retention

**Figure 3:**
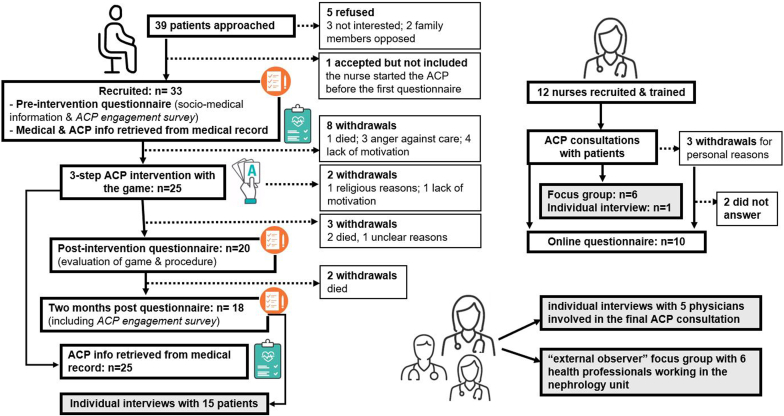
Flow diagram of recruitment and data collection. Boxes in grey represent the qualitative aspects of the study that are reported in a separate paper. ACP, advance care planning.

Patient recruitment took place from March 2022 to July 2023. Of the eligible patients, 87% (34/39) accepted to participate in the study. Sociodemographic and medical characteristics are described in Table 4. Of the included participants, 76% (25/33) started the 3-step ACP intervention, 70% (23/33) completed the ACP intervention, and 55% (18/33) completed the whole study procedure, including the 2-month postintervention questionnaire (Figure 3). Overall, 33% (5/15) of failed retention was due to premature death of patients. Reasons for withdrawal include anxiety about the subject (specifically about planning end-of-life), lack of trust in the medical team (mainly because of previous traumatic experiences), or lack of motivation (not convinced that ACP is important for them).

**Table 4:** Characteristics of participants, expressed in *n* (%) or mean ± SD, as appropriate

Characteristics	Patients *n* = 33	Nurses *n* = 10
Age (yrs)	67 ± 13	49 ± 7
Gender		
Female	10 (0.3)	10 (1)
Male	23 (0.7)	
Native language		
French	20 (0.6)	9 (0.9)
Other	13 (0.4)	1 (0.1)
Religious belief		
Christianism	22 (0.7)	5 (0.5)
Other religion	6 (0.2)	2 (0.2)
No religious belief	2 (0.2)	
No answer		3 (0.3)
Education (highest level achieved)		
Professional apprenticeship	13 (0.4)	
High school and vocational school	10 (0.3)	
University level	10 (0.3)	
Postgraduate training		
Chronic disease and renal failure (yrs)		10 (1)
Placed on transplant list		
Yes	7 (0.2)	
No	21 (0.6)	
In discussion	5 (0.2)	
Life place		
Home	32 (0.9)	
Nursing home	1 (0.1)	
Charlson score (gravity of illness and comorbidities)	8.3 ± 3.2	
Mode of dialysis		
Hemodialysis	27 (0.8)	
Peritoneal dialysis	6 (0.2)	
Patient working activity		
Yes	8 (0.2)	
No	24 (0.7)	
missing value	1	
At inclusion, supported by a close relative in the ACP process	26/30 (0.9) (3 missing data)	
Work rate (%)		78 ± 29
Number of yrs of experience		24 ± 10
Work unit		
Hemodialysis		4 (0.4)
Peritoneal dialysis		5 (0.5)
Both		1 (0.1)

ACP, advance care planning.

Nurse recruitment and training took place twice, in the beginning of 2022 and 2023. In total, 13 were interested, signed the protocol and completed the ACP training. They were all females experienced in nephrology care (Table 4). During the study, 2 nurses withdrew before using the game with a patient, and a third after having started (but not finished) the procedure with 3 patients. They provided a variety of reasons for their decision: ACP discussions with the game were deemed personally disturbing, too emotionally engaging, or too energy consuming. None was interested in participating in an individual interview.

### Acceptability and Feasibility

#### Evaluation of the Training

Overall, nurses evaluated the training program as positive, finding it adequate, supportive, and allowing them to master the intervention and use of the game (Table 3, TRAINING score). They all used the continuous training opportunity that was highly appreciated and allowed to identify barriers that are highlighted below.

#### Involvement of Relatives

Close relatives were strongly involved in the ACP process. At inclusion, 87% of patients reported having a close relative to support them in the process, and most patients who completed the 3-step ACP intervention (20/23), were accompanied by a close relative in ≥1 step of the procedure.

#### Evaluation of the 3-Step ACP Intervention

Both patients and nurses agreed that the intervention has a positive IMPACT: they reported that the intervention helped users to clarify their values and preferences related to ACP, express them, and plan related actions (Table 3). Notes taken by the research assistant while conducting the questionnaires indicate that some participants described the intervention as promoting humanity and trust within health care relationships.

**Figure 4:**
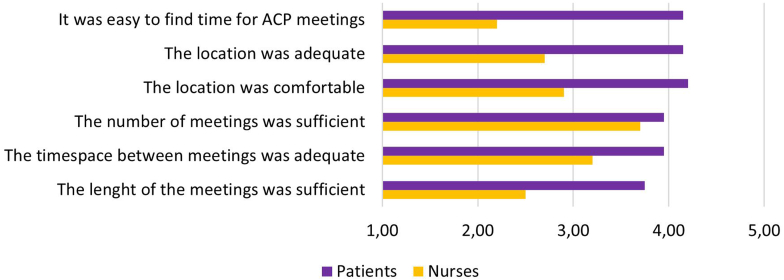
Nurses and patients’ mean answers to the 6 items of the organization score, ranging from 1 (not at all) to 5 (yes extremely). ACP, advance care planning.

As illustrated in Figure 4 and in Table 3, the ORGANISATION aspects of the intervention were more positively evaluated by patients (*n* =20, mean 6-items score = 4.06, SD = 0.4), than by nurses (*n* = 10, mean 6-items score = 2.87, SD = 0.49). The 1.19 between groups difference was statistically significant (*t* test: *t*(15.2) = −6.67, *P* ≤ 0.001, *d* = −2.67). In relation to this result, nurses provided additional qualitative feedback in the end-of-study questionnaire and during ongoing training supervision sessions (notes taken by the ACP coaches): notably, some nurses felt that ACP meetings competed with their other professional tasks or considered ACP discussions as an add-on supererogatory task. They also experienced difficulties in scheduling the ACP meetings within their working hours and to feel entitled to do so while their colleague nurses were overloaded with ordinary tasks. In addition, meeting scheduling was complex because of the number and diversity of participants, including patients’ relatives and physicians. Nurses worried about keeping track of what objectives had to be reached in each discussion session. Finally, some nurses expressed discomfort at conducting ACP counselling in common dialysis spaces with minimal curtain separations between beds and machinery noises.

In contrast, some participants reported to the research assistant that it was entertaining to use the game and conduct ACP discussions while undergoing boring routine dialysis sessions, it was positive to experience health care professionals’ interest in their priorities and values, and they did not mind others overhearing their discussions because they were not ashamed of their views and priorities.

#### Evaluation of the Card Game

Altogether, nurses (*n* = 10) and patients (*n* = 20) agreed that it is RELEVANT (Table 3) to use the game for ACP counselling and that the content of the cards is relevant. They evaluated the rules of the game and the content of the cards as easy to UNDERSTAND. They tended to find the topics of the cards more UPSETTING than INTRUSIVE. This highlights that the cards (some of which explicitly address end-of-life issues) can evoke strong emotions, but that this was considered acceptable.

**Figure 5:**
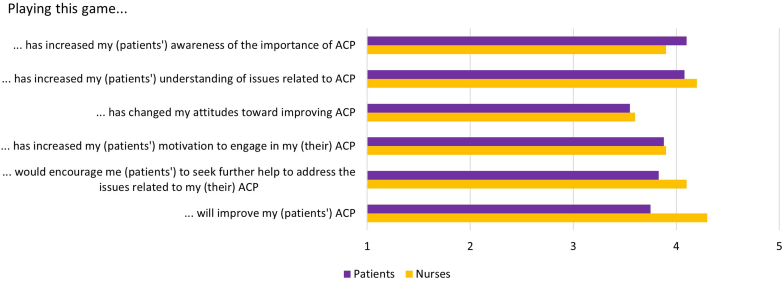
Patients’ and nurses’ mean answers, to the 6 items of the MARS-f scale, scoring on 1 to 5 scales (1 = strongly disagree; 5 = strongly agree). ACP, advance care planning.

Nurses and patients similarly scored high on the MARS-f subscale which measures the perception of the IMPACT OF THE GAME. They agreed that the game helps users become more aware of the importance of ACP and ADs, increases knowledge and positive attitude toward the topic, and that it encourages engagement in ACP behaviors (Figure 5).

**Figure 6:**
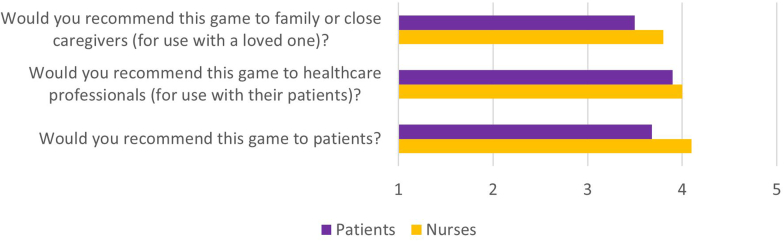
Patients’ and nurses’ mean answers, to 3 versions of the recommend question on a 1 to 5 scale: 1 = Not at all, I would not recommend this game to anyone; 2 = There are very few people I would recommend this game to; 3 = Maybe, there are a few people I would recommend this game to; 4 = There are many people I would recommend this game to; 5 = I would recommend this game to everyone.

Overall, patients and nurses endorsed the game, evaluating it with 4 out of 5 stars (Table 3, STAR EVALUATION) and recommending it to other users (Table 3, RECOMMENDATION & Figure 6).

### Effects of the Intervention

The intervention was followed by an important increase in uploaded ADs and health care surrogates named in patients’ medical records (Table 5). Furthermore, most participants reported in the postintervention questionnaire having done additional end-of-life planning actions (e.g. funerals, finance, will) during the time of the study.

**Table 5:** Pre-post effects of the 3-step ACP intervention

	At inclusion	At end of study	Statistics
	Number/number of responses (%)		
Advance directives (AD) in medical record	2/25 (8%)	10/25 (40%)	McNemar test: *P* = 0.005
Named health care surrogates in medical record	2/25 (8%))	23/25 (92%)	McNemar test: *P* < 0.001
Reported additional end-of-life planning actions		12/19	
	Averaged mean score on 1–5 scale ± SD (number of responses)		
Patients’ reported ENGAGEMENT in ACP: readiness and confidence to find a surrogate, to address ACP issues with surrogate or physician, and to write decisions in AD^a^ (1 = no, 5 = high ACP engagement)	3.22 ± 0.85 (*n* = 27) 3.04 ± 0.73 (*n* = 18)	4.08 ± 0.66 (*n* = 18)	+1.04 pre-post change (*n* = 18) Paired *t* test: *P* < 0.001
Overall quality of ACP DOCUMENTATION regarding (1) patients’ values, (2) end-of-life preferences, and (3) instructions for life-sustaining treatment (1 = no documentation, 5 = high quality documentation)	1.10 ± 0.4 (*n* = 33) 1.13 ± 0.46 (*n* = 25)	3.81 ± 1.06 (*n* = 25)	+2.68 pre-post change (*n* = 25) Paired *t* test: *P* < 0.001
Patients’ EVALUATION OF ACP DOCUMENTATION written in their record (1 = not at all, 5 = it fully corresponds to what they expressed during ACP consultations)		3.44 ± 1.04 (*n* = 18)	

AD, advance directive; ACP, advance care planning.

a Patients’ reported ENGAGEMENT was evaluated with the validated 9-item Advance Care Planning Engagement Survey.

**Figure 7:**
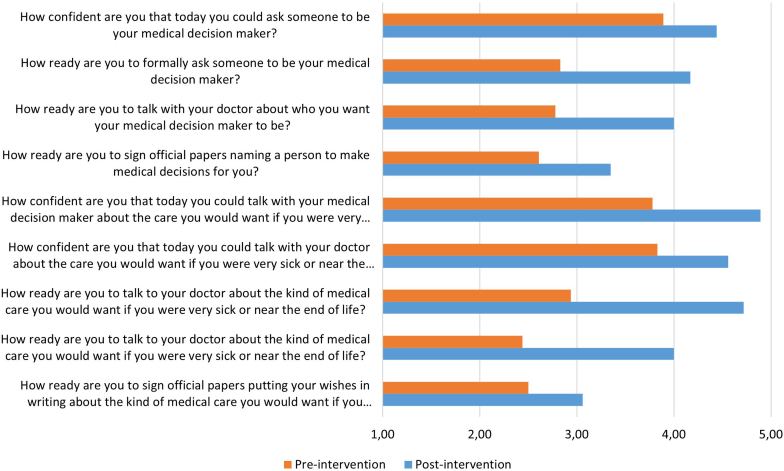
Patients’ mean answers, pre- and post-intervention, to the 9-items of the Advance Care Planning Engagement Survey, scoring on 1 to 5 scales (1 = not at all or I have never thought about it; 5 = extremely or I have already done it).

Patients reported a significant increase in ENGAGEMENT in ACP (paired *t* test: *t*(17) = 5.43, *P* ≤ 0.001, *d* =1.28). Specifically, results show a 1.04 increase in participants’ score on the ACP Engagement Survey (*n* = 18; before, mean 9-items score = 3.04, SD = 0.73; after, mean 9-items score = 4.08, SD = 0.66). Readiness to write ADs was less impacted than readiness and confidence to talk about ACP issues (Figure 7).

**Figure 8:**
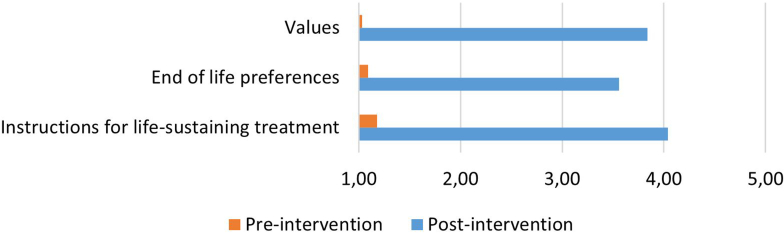
Quality of pre- and post-intervention advance care planning documentation in patients’ medical record, evaluated on a 1 to 5 score (1 = not at all clear; 5 = extremely clear).

A large pre-post change in the quality of ACP DOCUMENTATION (Table 5 and Figure 8) in medical records could be observed (paired *t* test: *t*(24) = 11.4, *P* ≤ 0.001, *d* = 2.28). Specifically, results show a large increase in mean documentation score (*n*= 25; before, mean = 1.13, SD=.09; after, mean = 3.81, SD = 0.21). Noteworthy, however, not all the content of the documentation was relevant or easy to translate into care. Some nurses for instance reported the title of the cards chosen by patients, which as such, provides little information about priorities for care. This difficulty was expressed during the continuous training supervision sessions (notes taken by the ACP coaches): some nurses reported being intimidated to write in patients’ medical records and having difficulties to translate patients’ view with medically relevant words.

Finally, overall patients’ EVALUATION OF THE ACP DOCUMENTATION written by nurses during the ACP procedure was good (*n* = 18; mean = 3.44, SD = 1.04), even though 2 patients were very critical (Table 5). Nevertheless, these patients evaluated the intervention and the game as positive.

## Discussion

Overall, patients and nurses evaluated as positive, the 3-step ACP intervention, the discussion tool *Anticip’action*, and the ACP training. All assessment measures related to acceptability obtained high mean scores, ranging from 2.9 to 4.2 on 1 to 5 scales (Table 3). This includes evaluation of the quality of the training, felt impact of the 3-steps intervention, evaluation of the impact and adequacy of the game, and overall recommendation of the game. Moreover, patients’ relatives were frequently involved in the process, actively participating in playing the game and in ACP discussions. These results confirm that the ACP intervention with *Anticip’action* can be well-received by target users. In contrast to other studies that have implemented ACP interventions with professionally trained champions,^8^^,^^10^^,^^29^ our study shows that it is possible to obtain similar acceptability results with a 10-hour training of ordinary nurses working in a medical unit.

Noteworthy however, we observed an important withdrawal rate. Three trained nurses (23%) withdrew before or after a first experience of ACP counselling, mainly because of the emotional burden. Nine participants (30%) who accepted to be included in the study later refused or persistently postponed the ACP consultations, for a variety of reasons (anxiety about end-of-life topics, lack of trust in the medical team, lack of interest). In contrast, notes taken by the research assistant indicate that some patients who accepted but without being fully convinced of the relevance of playing such a game, changed their mind and endorsed it after experiencing it. Overall, it means that ACP cannot be “imposed” as standard care, but could be standardly proposed in routine care, with a tactful communication strategy, leaving the opportunity to refuse. Similarly, ACP counselling should not be expected as a universal competence in care but could be attributed to specific nurses that feel at ease with end-of-life discussions.

Feasibility was evaluated as more problematic by health professionals than by patients. This was particularly visible in their differential responses to questions related to organization matters (Figure 4). Notably, nurses reported lack of time and difficulties in scheduling consultations and finding an adequate location, whereas patients did not experience those barriers. Similar barriers have been reported by health professionals in other studies,^9^^,^^10^^,^^30^ indicating the need to develop targeted strategies to alleviate them.

Several effects can be reported. The intervention was followed by a 30% increase in uploaded ADs and an 80% increase of designated health care surrogates in patients’ medical reports. Most patients (12/19) reported having completed additional end-of-life planning actions, indicating that *Anticip’action* had a direct action-motivating effect. Similar results were obtained in another study where the game was presented in a community setting.^22^ Moreover, a strikingly high 1.04 increase (on a 1–5 scale) in patients’ reported engagement in ACP could be observed, which is considerably higher than the 0.2 change usually considered as clinically meaningful.^31^^,^^32^ These results, however, highlight the incompressible proportion of patients (60%) that are not ready to write ADs despite having undergone (and mostly enjoyed) the 3-step ACP intervention (Figure 7 and Table 5). For these patients, ACP documentation written by health professionals in the medical record remains the only alternative.

With this regard, another effect of the 3-step intervention is the important pre-post increase (+ 2.68) in the quality of ACP documentation in patients’ medical records. This spectacular change is partly the result of a low baseline of ACP documentation at the beginning of the study, an explicit training provided to nurses, and repetitive instructions to document patients’ values and goals of care as part of the study intervention. To last over time, such good documentation practice needs to be actively entertained with training, supervision, and hierarchical support.

Importantly, this study integrated a novel assessment measure; patients were asked to verify and validate the ACP information documented by health care professionals in their medical record. Overall, they evaluated it as accurate (Table 5), which confirms the feasibility of the 3-step ACP intervention with *Anticip’action* and the importance to empower nurses and physicians to systematically create and maintain ACP documentation.

### Limitations

The study includes a small dataset, mostly composed of patients who completed the full procedure, which may tilt results toward positive outcomes. A selection bias is possible because participants who volunteered were likely more motivated and interested in ACP than the broader dialysis population.

This study was conducted in Geneva, a culturally diverse city, which supports some generalizability to multicultural populations. However, all participants were required to speak French (an inclusion criterion), which limited diversity. In addition, the study relied heavily on the support of the head of the nephrology unit. Therefore, the results may not fully apply to other countries or health care systems.

Some researchers were known to study participants, there were minor data reporting issues (detailed in Supplementary Results and Limitations), and qualitative data include researchers’ note taking, which may be impacted by researchers’ biases. Results of focus groups and interviews analyzed independently and published separately compensate for these limitations.

## Conclusion

The 3-step ACP intervention using the card game *Anticip’action* as a discussion tool was well-accepted by patients and nurses and had a relevant impact on patients’ engagement and on the quality of ACP documentation. It could be implemented in routine care, however, with a tactful communication strategy, leaving the opportunity to refuse. Organizational difficulties are barriers that need to be primarily addressed for successful implementation. Recent studies indicate that patients with chronic kidney disease and their families would appreciate starting ACP in early stages of the disease.^33^ It would be interesting to test the 3-steps procedure on this category of patients.

## Disclosure

All the authors declared no competing interests.

## Supplementary Material

Supplementary File (PDF) Supplementary Methods. Slides used for introducing the study protocol to nurses. Content of ACP training for nurses. Preintervention questionnaire for patients. Postintervention questionnaire for patients. Two months post-intervention questionnaire for patients. Information retrieved from participants’ medical records. End of study questionnaire for nurses. Supplementary Results. Limitations.
