# Supplementary material for: Advance Care Planning in Dialysis Patients With a Conversation Game
Source: Kidney Int Rep. 2025 Nov 10;11(2):103674. doi: 10.1016/j.ekir.2025.11.001 (PMC12769139; doi:10.1016/j.ekir.2025.11.001)
Supplement: Supplementary File (PDF) — Supplementary Methods. Slides used for introducing the study protocol to nurses. Content of ACP training for nurses. Preintervention questionnaire for patients. Postintervention questionnaire for patients. Two months post-intervention questionnaire for patients. Information retrieved from participants’ medical records. End of study questionnaire for nurses. Supplementary Results. Limitations. [file mmc1.pdf]

DOI: 10.1016/j.ekir.2025.11.001

Supplementary File

Contents

Material 1
Power point presentation used for introducing the study protocol to nurses 1
The game 1
Content of ACP training for nurses 2
Pre-intervention questionnaire for patients 2
Post-intervention questionnaire for patients 5
Two months post-intervention questionnaire for patients 8
Information retrieved from participants' medical records 9
End of study questionnaire for nurses 10
Methods 14
Translation of validated questionnaires 14
Score calculation & handling of missing values 14
Evaluation of the Quality of the Documentation 15
Statistical analysis 15
Results 16
Compliance to study procedures 16
Limitations 17

Material

Power point presentation used for introducing the study protocol to nurses

The slides contain 1) an explanation of the study procedure and of nurses' role within the study, and 2) useful background information related to ACP to take home.

The game

A light-coloured printable version of the game can be downloaded in English and French on the HUG website: https://www.hug.ch/centre-innovation/anticip-action

DOI: 10.1016/j.ekir.2025.11.001

Content of ACP training for nurses

**Activity**	**Duration**	**Content**
ACP e-learning	45’	- concepts and philosophy of ACP<br>- videos showing optimal *versus* non-optimal ways to initiate and conduct ACP conversations<br>- tutorial showing how to document ACP information in the patient record at the HUG (Geneva University Hospitals). In detail, there are three types of ACP documentation formats:<br>- patients’ advance directives that can be uploaded<br>- 5 checkbox options for providing ICU instructions in case of an urgent event<br>- open textbox to write patients’ values, goals of care, name of surrogate decision maker or any relevant ACP information
theoretical training	3h	- clarification of concepts such as ACP, surrogate decision makers, advance directives, instructions for intensive care units, goals of care<br>- legal aspects<br>- the three types of ACP documentation formats in patients’ HUG medical record<br>- how to identify patients that may benefit from ACP<br>- nurses’ expected competencies (knowledge, attitude, communication and relational skills)<br>- the 3-step ACP process including the game *Anticip’action*<br>- how to initiate the 3-step process with a patient
2 X practical training sessions	3h each	- recapitulation of 3-step ACP procedure (see description in the article, Table 3) & rules of the game *Anticip’action*<br>- practical training of the 3-step ACP intervention with patients-as-partners: for each step, 30’ to 45’ role-playing in groups of 2 or 3 persons (roles played = nurse, patient or external observer)<br>- feedback on nurses’ posture and attitude<br>- identification of what ACP information to write in patients’ HUG medical record & writing training<br>- clarification of nurses’ role in the study research protocol (what is expected from them and why)
5 X continuous training sessions	1h each	- feedback and exchange meetings with two experienced ACP coaches<br>- discussions about barriers and facilitators<br>- help to resolve complex situations

Pre-intervention questionnaire for patients

	**Sociodemographic information**	
1	Quel est votre pays de naissance ?<br>*What is your country of birth?*	liste déroulante de pays<br>*country drop-down list*
2	Quelle est votre langue de naissance ?<br>*What is your native language?*	Liste déroulante de langues<br>*Language drop-down list*
3	Quelles sont vos croyances ou appartenances religieuses ?<br>*What are your beliefs or religious affiliations?*	Christianisme, Islam, Judaïsme, Bouddhisme, Hindouisme, Animisme, autre, sans, ne veut pas répondre<br>*Christianity / Islam / Judaism / Buddhism / Hinduism / Animism / other / without / doesn't want to answer*
4	Quel est le plus haut niveau de formation que vous avez atteint ?<br>*What is the highest level of education you have reached?*	Aucune formation / Ecole primaire (élémentaire) / Ecole professionnelle (apprentissage) / Gymnase / Maturité professionnelle / Haute école (Hes) ou école spécialisée / Université

		No formal education grade / Primary School (Elementary) Vocational School (apprenticeship) Gymnasium / Vocational Baccalaureate University of Applied Sciences (HES) or Specialized School University
5	Est-ce que vous avez un travail rémunéré en ce moment ?<br>*Do you have a paid job at the moment?*	Oui / non<br>*Yes / no*
	**Items of ACP Engagement Survey ©***<br>©These questions are taken from the validated questionnaire distributed on the *Prepare for your care* website (https://prepareforyourcare.org/research), licensed under the Creative Commons Attribution-NonCommercial-ShareAlike 4.0 International License. https://creativecommons.org/licenses/by-nc-sa/4.0/ © 2013 The Regents of the University of California, www.prepareforyourcare.org<br>Questions 1-9 correspond to the validated 9-item version of the questionnaire. Questions 10 and 11 have been selected from the 34-item version of the questionnaire and correspond to questions 33 and 34 exploring the theme: “Asking your doctor questions”. We added them because we thought that they were particularly relevant to our study. At analysis stage, it appeared that adding these questions did not make any relevant score difference compared to the 9-item version. They therefore are not reported in the paper. These questions were translated according to the good practices described in Wild et al. 2005.	
	Nous allons vous poser des questions sur vos expériences et vos opinions. Il se peut que nos questions concernent des choses que vous avez déjà faites ou auxquelles vous n'avez jamais pensé. Répondez de la manière la plus honnête possible.<br><br>*Nous vous interrogerons sur 3 sujets:*<br><br>*Le ou la représentant.e thérapeutique, ou remplaçant.es*<br>*Les décisions sur ce qui importe le plus dans la vie*<br>*Poser des questions aux médecins*<br><br>*Les premières questions concernent le ou la représentant.e thérapeutique. Un ou une représentant.e thérapeutique est un membre de la famille ou un.e ami.e qui peut prendre des décisions à votre place si vous devenez trop malade pour le faire vous-même.*<br><br>*N'oubliez pas, l'important ici est de répondre le plus honnêtement possible. Il n'y a pas de bonnes ou de mauvaises réponses.*	
1	Dans quelle mesure vous sentez-vous en confiance aujourd'hui pour demander à quelqu'un d'être votre représentant.e thérapeutique?	1. Pas du tout / 2. Un peu / 3. Quelque peu / 4. Assez / 5. Extrêmement / 8. Pas sûr.e / 9. Refuse de répondre
2	Dans quelle mesure êtes-vous prêt.e à demander officiellement à quelqu'un d'être votre représentant.e thérapeutique ?	1. Je n'y ai jamais pensé / 2. J'y ai pensé, mais je ne suis pas prêt.e à le faire / 3. Je pense le faire dans les prochains 6 mois / 4. J'ai certainement l'intention de le faire dans les 30 prochains jours / 5. Je l'ai déjà fait / 8. Je ne suis pas sûr.e / 9. Je refuse de répondre<br><br>OPTIONEL : Si la personne a répondu "Je l'ai déjà fait", demandez-lui "Quand l'avez-vous fait ?"<br>1. Il y a moins de 6 mois / 2. Il y a plus de 6 mois / 8. Pas sûr.e / 9. Refuse de répondre
3	Dans quelle mesure êtes-vous prêt.e à parler avec votre médecin de qui vous souhaiteriez comme représentant.e thérapeutique?	‘’
4	Dans quelle mesure êtes-vous prêt.e à signer un document officiel nommant une personne ou ses remplaçant.es pour	‘’

	prendre des décisions médicales à votre place?	
	Nous allons maintenant changer de sujet. Les questions suivantes portent sur des traitements médicaux spécifiques que les gens pourraient accepter ou refuser s'ils étaient très malades ou en fin de vie. Par exemple, certaines personnes savent qu'elles accepteraient d'être placées sous respirateur (d'être aidées par des machines pour respirer). D'autres savent qu'elles ne voudraient jamais être placées sous respirateur. Veuillez répondre en toute honnêteté aux questions suivantes concernant les traitements médicaux. Il n'y a pas de bonnes ou de mauvaises réponses.<br><br>*Les deux prochaines questions visent à déterminer dans quelle mesure vous vous sentez en confiance pour parler à quelqu'un de vos souhaits en matière médicale.*	
5	Dans quelle mesure vous sentez-vous en confiance aujourd'hui pour parler à votre REPRESENTANT.E THERAPEUTIQUE des soins que vous souhaiteriez recevoir si vous étiez très malade ou en fin de vie ?	1. Pas du tout / 2. Un peu / 3. Quelque peu / 4. Assez / 5. Extrêmement / 8. Pas sûr.e / 9. Je refuse de répondre
6	Dans quelle mesure vous sentez-vous en confiance aujourd'hui pour parler à votre MEDECIN des soins que vous souhaiteriez recevoir si vous étiez très malade ou en fin de vie ?	"
	Les deux prochaines questions visent à déterminer dans quelle mesure vous vous sentez en confiance pour parler à quelqu'un de vos souhaits en matière médicale.	
7	Dans quelle mesure êtes-vous prêt.e à parler à votre REPRESENTANT.E THERAPEUTIQUE du type de soins médicaux que vous souhaiteriez recevoir si vous étiez très malade ou en fin de vie ?	1. Je n'y ai jamais pensé / 2. J'y ai pensé, mais je ne suis pas prêt.e à le faire / 3. Je pense le faire dans les prochains 6 mois / 4. J'ai certainement l'intention de le faire dans les 30 prochains jours / 5. Je l'ai déjà fait / 8. Je ne suis pas sûr.e / 9. Je refuse de répondre<br><br>OPTIONEL : Si la personne a répondu "Je l'ai déjà fait", demandez-lui "Quand l'avez-vous fait ?"<br>1. Il y a moins de 6 mois / 2. Il y a plus de 6 mois / 8. Pas sûr.e / 9. Refuse de répondre
8	Dans quelle mesure êtes-vous prêt.e à parler à votre MEDECIN du type de soins médicaux que vous souhaiteriez recevoir si vous étiez très malade ou en fin de vie ?	"
9	Dans quelle mesure êtes-vous prêt.e à SIGNER UN DOCUMENT OFFICIEL indiquant vos souhaits concernant le type de soins médicaux que vous souhaiteriez recevoir si vous étiez très malade ou en fin de vie ?	"
	Nous allons maintenant parler des questions que vous pourriez poser aux médecins.	
10	Dans quelle mesure vous sentez-vous en confiance aujourd'hui pour poser les bonnes questions à votre médecin pour vous aider à prendre de bonnes décisions médicales?	1. Pas du tout / 2. Un peu / 3. Quelque peu / 4. Assez / 5. Extrêmement / 8. Pas sûr.e / 9. Je refuse de répondre
11	Dans quelle mesure êtes-vous prêt.e à poser des questions à votre médecin pour vous aider à prendre une bonne décision en matière médicale?	1. Je n'y ai jamais pensé / 2. J'y ai pensé, mais je ne suis pas prêt.e à le faire / 3. Je pense le faire dans les prochains 6 mois / 4. J'ai certainement l'intention de le faire dans les 30 prochains jours / 5. Je l'ai déjà fait / 8. Je ne suis pas sûr.e / 9. Je refuse de répondre

		OPTIONEL : Si la personne a répondu "Je l'ai déjà fait", demandez-lui "Quand l'avez-vous fait ?"<br>1. Il y a moins de 6 mois / 2. Il y a plus de 6 mois / 8. Pas sûr.e / 9. Refuse de répondre

Post-intervention questionnaire for patients

Consigne : Dans le cadre de l'étude ACP, vous avez rencontré plusieurs fois l'infirmier-(ère) pour discuter de vos valeurs, priorités et objectifs en rapport avec vos soins et votre vie. Nous aimerions connaître votre évaluation concernant la planification et le contexte de ces discussions et concernant le jeu Anticip'action que vous avez utilisé. Pour chaque question, veuillez choisir une option de réponse.<br>*Instructions: As part of the ACP study, you met with the nurse several times to discuss your values, priorities and goals related to your care and life. We would like to hear your assessment of the planning and context of these discussions and of the game Anticip'action you used. For each question, please choose an answer option.*		
	**Regarding the ACP intervention**	
1	C'était facile de trouver du temps pour ces interventions de projet de soins anticipé.<br>*It was easy to find time to complete the ACP process*	Pas du tout d'accord/ Pas d'accord/ Ni en désaccord, ni d'accord / D'accord / Tout à fait d'accord / Je ne sais pas quoi répondre / Je préfère ne pas répondre<br>*Strongly disagree/ Disagree/ Neither disagree nor agree / Agree / Strongly agree / I don't know what to answer / I prefer not to answer*
2	Le lieu des rencontres était adéquat<br>*The room used for the ACP was adequate*	''
3	Le lieu des rencontres était confortable<br>*The place of the meetings was comfortable*	''
4	Le nombre de rencontres était suffisant<br>*The number of meetings scheduled for the intervention was sufficient*	''
5	L'espacement des rencontres était adéquat<br>*The spacing of the meetings was adequate*	''
6	Le temps proposé était suffisant<br>*The length of the meetings was sufficient*	''
8	Cette démarche de projet de soins anticipé en trois séances m'a permis de *clarifier* ce qui m'importe le plus (mes valeurs, préférences, objectifs de soin)<br>*This 3-step ACP process helped CLARIFY what is most important to me (values, preferences, goals of care)*	''
9	Cette démarche de projet de soins anticipé en trois séances m'a permis *d'exprimer* ce qui m'importe le plus (mes valeurs, préférences, objectifs de soin)<br>*This 3-step ACP process helped to EXPRESS what is most important to me (values, preferences, goals of care)*	''
10	Cette démarche de projet de soins anticipé en trois séances m'a permis de *planifier des actions concrètes* (par ex. avoir une discussion avec une personne proche, organiser mon futur, écrire mon testament)<br>*This 3-step ACP process helped to plan actions (e.g. having a specific discussion with a close*	''

	person, organizing my future in a concrete way, writing my will, etc.)	
	**Regarding the game *Anticip'action***	
1	J'ai trouvé pertinent d'utiliser le jeu Anticip'action avec un.e soignant.e pour soutenir mon projet de soins anticipé<br>*I found it relevant to use the game Anticip'action with a nurse in order to conduct my ACP*	"
2	J'ai trouvé les règles du jeu faciles à comprendre<br>I found the rules of the game easy to understand	"
3	J'ai trouvé les questions/énoncés (sur les cartes) faciles à comprendre<br>*I found the questions (words on the cards) easy to understand*	"
4	J'ai trouvé les questions/énoncés pertinentes<br>*I found the questions (words on the cards) relevant*	"
5	J'ai trouvé les questions/énoncés bouleversantes<br>*I found the questions (words on the cards) easy to understand upsetting*	"
6	J'ai trouvé les questions/énoncés intrusives<br>*I found the questions (words on the cards) intrusive*	"
6bis	<Si oui à Q6> Quelle(s) question(s) étai(en)t intrusive(s) et pourquoi ?<br>*<If yes to Q6> Which question(s) were intrusive and why?*	Champ texte<br>*Text field*
7	J'ai utilisé une (ou plusieurs) carte joker ou un (ou plusieurs) thème qui m'importe ne se trouvait pas sur les cartes.<br>*I used one (or more) joker cards or a theme (or several) that matters to me was not on the cards.*	Oui/non<br>*Yes/No*
7bis	<Si oui à Q7> Pouvez-vous en dire plus sur ce(s) thème(s) ?<br>*<If yes to Q7> Can you tell us more about this topic(s)?*	Champ texte<br>*Text field*
8*©	Jouer à ce jeu m'a fait prendre conscience de l'importance du projet de soins anticipé (c'est-à-dire du processus consistant à définir, discuter et consigner mes objectifs et mes préférences en matière de traitement et de soins médicaux futurs).<br>*Playing this game has increased my awareness of the importance of advance care planning (that is, the process of defining, discussing and recording my goals and preferences for future medical treatment and care)*	Pas du tout d'accord / Pas d'accord / Ni en désaccord, ni d'accord / D'accord / Tout à fait d'accord / Je ne sais pas quoi répondre / Je préfère ne pas répondre<br>*Strongly disagree / Disagree / Neither disagree nor agree / Agree / Strongly agree / I don't know what to answer / I prefer not to answer*
9*©	Jouer à ce jeu m'a permis de mieux connaître/comprendre les questions liées au projet de soins anticipé.<br>*Playing this game has increased my knowledge/understanding of issues related to advance care planning*	".
10*©	Jouer à ce jeu a changé mon attitude face au projet de soins anticipé.	"

	Playing this game has changed my attitudes toward improving advance care planning	
11*©	Jouer à ce jeu a augmenté mon intention et ma motivation à m'engager dans mon projet de soins anticipé<br>*Playing this game has increased my intentions/motivation to engage in my advance care planning*	"
12*©	Jouer à ce jeu peut m'encourager à chercher plus d'aide pour répondre aux questions relatives à mon plan de soins anticipé (au besoin)<br>*Playing this game would encourage me to seek further help to address the issues related to my advance care planning (if I needed it)*	"
13*©	Jouer à ce jeu améliorera mon projet de soins anticipé<br>*Playing this game will improve my advance care planning*	"
14*©	Quel est votre classement général (par étoiles) de ce jeu ?<br>*What is your overall (star) rating of the game?*	* Inadéquat / **pas bon / ***acceptable / ****bon / *****excellent<br>** Inadequate / **not good / ***acceptable / ****good / *****excellent*
15*©	Recommanderiez-vous ce jeu à d'autres patients?<br>*Would you recommend this game to other patients?*	1. Pas du tout. Je ne recommanderais ce jeu à personne. / 2. Il y a très peu de gens à qui je recommanderais ce jeu. / 3. Peut-être. Il y a plusieurs personnes à qui je recommanderais ce jeu. / 4. Il y a beaucoup de personnes à qui je recommanderais ce jeu. /5. Je recommanderais ce jeu à tout le monde.<br>*1. Not at all. I wouldn't recommend this game to anyone. / 2. There are very few people I would recommend this game to. / 3. Maybe. There are several people I would recommend this game to. / 4. There are many people I would recommend this game to. / 5. I would recommend this game to everyone.*
16*©	Recommanderiez-vous ce jeu aux professionnels de la santé (pour l'utiliser avec leurs patient.es) ?<br>*Would you recommend this game to healthcare professionals (for use with their patients)?*	"
17*©	Recommanderiez-vous ce jeu aux proches aidant.es (pour l'utiliser avec un être cher)?<br>*Would you recommend this game to family caregivers or close caregivers (for use with a loved one)?*	"
18*	J'ai aussi utilisé le jeu Anticip'action en dehors des deux consultations avec mon.ma soignant.e<br>*I have used the game in addition to ACP consultations with the nurse (e.g. at home)*	Oui, avec plusieurs personnes / Oui, avec une personne / Oui mais j'ai joué seul.e / Non, je n'ai pas joué / Je ne sais pas quoi répondre / Je préfère ne pas répondre<br>*Yes, with several people / Yes, with one person / Yes but I played alone / No, I didn't play / I don't know what to answer / I prefer not to answer*
19*	J'ai consulté l'application *Accordons-nous* (module de Concerto)	1. Oui très souvent / 2. Oui, à plusieurs reprises et avec attention / 3. Pas très souvent mais avec application quand je l'ai fait / 4.

	I have used the app Accordons-nous (included as module in the app Concerto)	Seulement une ou deux fois rapidement / 5. Je n'ai pas vraiment réussi ou trouvé l'occasion de le faire<br>1. Yes very often / 2. Yes, repeatedly and carefully /3. Not very often but diligently when *I did it / 4. Only once or twice quickly / 5. I didn't really succeed or find the opportunity to do it*<br><br>Si réponse 5 : Pourquoi ? → radio bouton: - parce que cette application ne m'intéresse pas - parce que je ne suis pas très à l'aise avec un outil numérique - parce que je ne connais pas cette application - autre + texte ouvert<br>*If answer 5: Why? radio button options: - because this application does not interest me - because I'm not very comfortable with a digital tool - because I don't know this app - Other + Open Text*<br><br>Si réponse 1, 2, 3 ou 4 : Quand l'avez-vous consultée (plusieurs réponses possibles) ? → cases à cocher: - avant de participer à cette étude / - durant une des deux séances avec le jeu Anticip'action / - durant la 3ème séance (consultation avec le.la médecin) / - en privé entre les 3 séances de l'intervention / - après la 3ème séance avec le médecin<br>*If answer 1, 2, 3 or 4: When did you consult it (several possible answers)? boxes to tick: - before participating in this study / - during one of the two sessions with the Anticip'action game / - during the 3rd session (consultation with the doctor) / - in private between the 3 sessions of the procedure / - after the 3rd session with the doctor*

* These questions were asked twice: after having played the game and 2 months after the last session of the multi-step intervention. © These questions are taken (and adapted) from the validated questionnaire published in Stoyan R Stoyanov, Leanne Hides, David J Kavanagh, Hollie Wilson. Originally published in JMIR Mhealth and Uhealth (http://mhealth.jmir.org), 10.06.2016. It is distributed under the terms of the Creative Commons Attribution License (http://creativecommons.org/licenses/by/2.0/). These questions were translated according to the good practices described in Wild et al. 2005.

Two months post-intervention questionnaire for patients

	ACP-Engagement survey items (see pre-intervention questionnaire for patients)	5 points scales
	Questions marked with * in the Post-intervention questionnaire for patients	5 points scales
1	Aviez-vous déjà écrit vos directives anticipées AVANT d'entrer dans cette étude ?<br>*Had you already written your advance directives BEFORE entering this study?*	Oui / Non / Je ne me souviens plus / Je ne sais pas quoi répondre / Je préfère ne pas répondre<br>*Yes / No / I don't remember / I don't know*

		what to answer / I prefer not to answer
1bis	[*Si le.la patient.e a répondu « oui » à la question précédente*] Depuis votre entrée dans cette étude, avez-vous vérifié le contenu de vos directives anticipées ou les avez-vous remis à jour ?<br>*[If the patient answered "yes" to the previous question] Since you joined this study, have you checked the content of your advance directives or have you updated them?*	Oui / Non + Je ne sais pas quoi répondre + Je préfère ne pas répondre<br>*Yes / No / I don't know what to answer / I prefer not to answer*
2	Depuis que vous avez joué avec Anticip'action, avez-vous réalisées une/plusieurs actions concrètes ? (avoir une discussion particulière avec une personne proche, organiser votre futur de manière concrète, écrire votre testament, etc.)<br>*Since you played with the game Anticip'action, have you carried out one or more concrete actions? (e.g. having a specific discussion with someone close to you, organizing your future in a concrete way, writing your will, etc.).*	Oui, j'ai réalisé plusieurs actions / Oui, j'ai réalisé une action / Non / Je ne sais pas quoi répondre / Je préfère ne pas répondre<br>*Yes, I have performed several actions / Yes, I have performed an action / No / I don't know what to answer / I prefer not to answer*
3	Ce qui est écrit dans la rubrique « Projet de soins anticipé » dans mon dossier patient correspond à ce que j'ai exprimé lors des entretiens en lien avec le jeu.<br>*The written documentation in my medical record corresponds to what I expressed during the ACP consultations in connection with the game.*	Pas du tout d'accord/ pas d'accord/ ni en désaccord, ni d'accord / d'accord / tout à fait d'accord / Je ne sais pas trop / Je préfère ne pas répondre<br>*Strongly disagree / disagree / neither agree nor disagree / agree / strongly agree / not sure / prefer not to answer*

Information retrieved from participants' medical records

1	sociodemographic information	- age (nb)<br>- gender (woman, man, other)<br>- living sites (at home / in an elderly care institution / in another healthcare structure, other (open text))
2	Medical information	**Nephropathy**:(one answer only)<br>- Vascular nephropathy<br>- Diabetic nephropathy<br>- Vascular and diabetic nephropathy<br>- Chronic glomerulonephritis<br>- Kystic diseases<br>- Others<br>**Comorbidities**: (multiple answers possible)<br>- Hypertension<br>- Diabetes mellitus<br>- Ischemic heart disease<br>- Peripheral vascular disease<br>- Previous cerebrovascular event<br>- Cognitive impairment<br>- Assistance required when walking<br>- None

		- Other (open text)<br>**Type of dialysis**<br>- Hemodialysis (HD)<br>o If yes, "since when? (date)<br>- Peritoneal Dialysis (DP)<br>o If yes, "since when? (date)<br>**In dialysis since**: date<br>**On transplant list**<br>- Undergoing transplant assessment<br>- No<br>- Yes → "since when? (date)<br>**CharlsonScore**
3*	Content of ACP section	- Advanced directives uploaded? (no / yes)<br>→ if yes, last update<br>- Medical decision maker designated? (no / yes)<br>→ if yes, last update<br>- life-sustaining treatment (IMU) completed (no / yes)<br>→ if yes: last update & IMU classification (IMU-1, IMU2, IMU3, IMU4, IMU5)<br>- documented discussion about values or goals (no / yes)<br>→ if yes: last update & clarity evaluation (1= Not at all clear ; 2= A little clear ; 3= Somewhat clear ; 4= Fairly clear ; 4 = Extremely clear)***<br>- documented discussion about end-of-life care planning (no / yes)<br>→ if yes: last update & clarity evaluation (1= Not at all clear ; 2= A little clear ; 3= Somewhat clear ; 4= Fairly clear ; 4 = Extremely clear)***<br>- documented discussion about life-sustaining treatment preferences (no / yes)<br>→ if yes: last update & clarity evaluation (1= Not at all clear ; 2= A little clear ; 3= Somewhat clear ; 4= Fairly clear ; 4 = Extremely clear)***
4**	Number of updates in the ACP section since the inclusion of the participant in the study	- Nb of updates in the field IMU (nb)<br>- Nb of new upload of DA (nb)<br>- Nb of updates in the other ACP fields (nb)

* Information retrieved at the pre-intervention stage and at the 2-month post-intervention stage.

** Information retrieved only once at the 2-month post-intervention stage.

End of study questionnaire for nurses

	**Sociodemographic information**	
1	Quel est votre pays de naissance ?<br>*What is your country of birth?*	liste déroulante de pays<br>*country drop-down list*
2	Quelle est votre langue de naissance ?<br>What is your native language?	Liste déroulante de langues<br>Language drop-down list
3	Quelles sont vos croyances ou appartenances religieuses ?<br>*What are your beliefs or religious affiliations?*	Christianisme, Islam, Judaïsme, Bouddhisme, Hindouisme, Animisme, autre, sans, ne veut pas répondre<br>*Christianity / Islam / Judaism / Buddhism / Hinduism / Animism / other / without / doesn't want to answer*
4	Quel est votre âge ?<br>*What is your age ?*	nb
5	Quel est votre genre ?	Femme, homme, autre

	What is your gender ?	*Female, male, other*
6	Quel est votre taux de travail ?<br>*What is your percentage of work activity?*	nb
7	Combien d'années d'expérience professionnelle en tant qu'infirmières avez-vous ?<br>*How many years of professional experience as a nurse do you have?*	nb
8	Quel est votre niveau de spécialisation professionnelle ?<br>*What is your level of professional specialization?*	Cases à cocher : - CAS (ou équivalent) / - DAS (ou équivalent) / - Master (ou équivalent) / - Autre<br>*Checkboxes: CAS (or equivalent) / - DAS (or equivalent) / - Master (or equivalent) / - Other*
9	Précision sur votre spécialisation professionnelle<br>*More information related to your professional specialization*	Champ texte<br>*Text field*
10	Dans quel service travaillez-vous ?<br>*In which unit do you work?*	Hémodialyse / Dialyse péritonéale / Les deux<br>*Hemodialysis / Peritoneal Dialysis / Both*
	**Regarding training and supervision**	
1	Le contenu de la formation est adéquat<br>*The content of the training is adequate*	Pas du tout d'accord/ pas d'accord/ ni en désaccord, ni d'accord / d'accord / tout à fait d'accord<br>*Strongly disagree/ disagree/ nor disagree nor agree / agree / strongly agree*
2	Les espaces de débriefing et de supervision m'ont permis de clarifier le protocole<br>*The training helped to understand the study protocol*	"
3	Je me suis sentie soutenue<br>*I felt supported*	".
4	A la fin de la formation, je maitrisais l'utilisation du jeu<br>*At the end of the training, I mastered the use of the game*	"
	**Regarding the ACP intervention**	
1	C'était facile de trouver du temps pour ces interventions pour mon projet de soins anticipé<br>*It was easy to find time to complete the ACP process*	"
2	Le lieu des rencontres était adéquat<br>*The room used for the ACP was adequate*	"
3	Le lieu des rencontres était confortable<br>The place of the meetings was comfortable	"
4	Le nombre de rencontres était suffisant<br>*The number of meetings scheduled for the intervention was sufficient*	"
5	L'espacement des rencontres était adéquat<br>*The spacing of the meetings was adequate*	"
6	Le temps proposé était suffisant<br>*The length of the meetings was sufficient*	"
8	Cette démarche de projet de soins anticipé en trois séances a permis à mes patients de *clarifier* ce qui	"

	leur importe le plus (leurs valeurs, préférences, objectifs de soin)<br>*This 3-step ACP process helped my patients to CLARIFY what is most important to me (values, preferences, goals of care)*	
9	Cette démarche de projet de soins anticipé en trois séances a permis à mes patients *d'exprimer* ce qui leur importe le plus (leurs valeurs, préférences, objectifs de soin)<br>*This 3-step ACP process helped my patients to EXPRESS what is most important to me (values, preferences, goals of care)*	"
10	Cette démarche de projet de soins anticipé en trois séances a permis à mes patients de *planifie*r des actions concrètes (par ex. avoir une discussion avec une personne proche, organiser leur futur, écrire leur testament)<br>*This 3-step ACP process helped my patients to plan actions (e.g. having a specific discussion with a close person, organizing my future in a concrete way, writing my will, etc.)*	"
11	Combien de patients avez-vous pris en charge dans le cadre de ce protocole de recherche ?<br>*How many patients have you taken care of as part of this research protocol?*	nb
12	J'ai bien suivi le protocole avec mes patients<br>*I followed the study protocol well with my patients*	Pas du tout d'accord/ pas d'accord/ ni en désaccord, ni d'accord / d'accord / tout à fait d'accord<br>*Strongly disagree/ disagree/ nor disagree nor agree / agree / strongly agree*
	**Regarding the game *Anticip'action***	
1	J'ai trouvé pertinent d'utiliser le jeu Anticip'action avec mes patient.es pour soutenir leur projet de soins anticipé<br>*I found it relevant to use the game Anticip'action with my patients in order to conduct their ACP*	".
2	J'ai trouvé les règles du jeu faciles à comprendre<br>*I found the rules of the game easy to understand*	"
3	J'ai trouvé les questions/énoncés (sur les cartes) faciles à comprendre<br>*I found the questions (words on the cards) easy to understand*	"
4	J'ai trouvé les questions/énoncés pertinentes<br>*I found the questions (words on the cards) relevant*	"
5	J'ai trouvé les questions/énoncés bouleversantes<br>*I found the questions (words on the cards) easy to understand upsetting*	"
6	J'ai trouvé les questions/énoncés intrusives<br>*I found the questions (words on the cards) intrusive*	"
6bis	<Si oui à Q6> Quelle(s) question(s) étai(en)t intrusive(s) et pourquoi ?<br>*<If yes to Q6> Which question(s) were intrusive and why?*	Champ texte<br>*Text field*

7	Les patients ont utilisé une (ou plusieurs) carte joker ou ont évoqué des thèmes qui n'étaient pas sur les cartes.<br>*The patients used one (or more) joker cards or a theme (or several) that mattered to them was not on the cards.*	Oui/non<br>*Yes/no*
7bis	<Si oui à Q7> Pouvez-vous en dire plus sur ce(s) thème(s) ?<br>*<If yes to Q7> Can you tell us more about this topic(s)?*	Champ texte<br>*Text field*
8	Jouer à ce jeu a fait prendre conscience à mes patients de l'importance du projet de soins anticipé (c'est-à-dire du processus consistant à définir, discuter et consigner les objectifs et préférences en matière de traitement et de soins médicaux futurs).<br>*Playing this game has increased my patients' awareness of the importance of advance care planning (that is, the process of defining, discussing and recording my goals and preferences for future medical treatment and care)*	Pas du tout d'accord/ pas d'accord/ ni en désaccord, ni d'accord / d'accord / tout à fait d'accord<br>*Strongly disagree/ disagree/ nor disagree nor agree / agree / strongly agree*
9	Jouer à ce jeu a permis à mes patients de mieux connaître/comprendre les questions liées au projet de soins anticipé.<br>*Playing this game has increased my patients' knowledge/understanding of issues related to advance care planning*	''.
10	Jouer à ce jeu a changé l'attitude de mes patients face au projet de soins anticipé.<br>*Playing this game has changed my patients' attitudes toward improving advance care planning*	''
11	Jouer à ce jeu a augmenté l'intention et la motivation de mes patients à s'engager dans leur projet de soins anticipé<br>*Playing this game has increased my patients' intentions/motivation to engage in their advance care planning*	''
12	Jouer à ce jeu peut encourager mes patients à chercher plus d'aide pour répondre aux questions relatives à leur plan de soins anticipé (au besoin)<br>*Playing this game would encourage my patients to seek further help to address the issues related to their advance care planning (if they needed it)*	''
13	Jouer à ce jeu améliorera le projet de soins anticipé de mes patients<br>*Playing this game will improve my patients' advance care planning*	''
14	Quel est votre classement général (par étoiles) de ce jeu ?<br>*What is your overall (star) rating of the game?*	* Inadéquat / **pas bon / ***acceptable / ****bon / *****excellent<br>** Inadequate / **not good / ***acceptable / ****good / *****excellent*
15	Recommanderiez-vous ce jeu à d'autres patients?<br>*Would you recommend this game to other patients?*	1. Pas du tout. Je ne recommanderais ce jeu à personne. / 2. Il y a très peu de gens à qui je recommanderais ce jeu. / 3. Peut-être. Il y a plusieurs personnes à qui je recommanderais ce jeu. / 4. Il y a beaucoup de personnes à qui

		je recommanderais ce jeu. /5. Je recommanderais ce jeu à tout le monde.<br>*1. Not at all. I wouldn't recommend this game to anyone. / 2. There are very few people I would recommend this game to. / 3. Maybe. There are several people I would recommend this game to. / 4. There are many people I would recommend this game to. / 5. I would recommend this game to everyone.*
16	Recommanderiez-vous ce jeu aux professionnels de la santé (pour l'utiliser avec leurs patient.es) ?<br>*Would you recommend this game to healthcare professionals (for use with their patients)?*	"
17	Recommanderiez-vous ce jeu aux proches aidant.es (pour l'utiliser avec un être cher)?<br>*Would you recommend this game to family caregivers or close caregivers (for use with a loved one)?*	"
18	Pour terminer, vous pouvez ajouter ici toutes vos remarques ou commentaires en rapport avec la démarche et le jeu<br>*Finally, you can add here all your remarks or comments related to the approach and the game*	Champ texte<br>*Text field*

Methods

Translation of validated questionnaires

Items from two English validated questionnaires: the *ACP engagement survey* [21] and the *user MARS questionnaire, section f* [22] were translated in French and adapted to the relevant cultural context following the good practices described in Wild et all [23]. Two independent English-French forward translations were ordered. An expert committee composed of all authors reconciled the translations. A professional back translation was then ordered. The expert committee reviewed the back translation against the source questionnaire and harmonized the French version that was pilot-tested with 7 users from the general and target population. This review process allowed to finalize the translation.

Score calculation & handling of missing values

Whenever possible, 1-5 scale items were used. Questions addressing the same topics were pooled together to create overall scores. All overall scores were calculated as averages of responses to their items.

The dataset contains occasional missing values on the 1-5 scale either because the study nurse did not fill in one question (unidentified reason) or because the participants answered “I do not know” or “I do not wish to answer” to the research assistant. If no more than one value was missing for

calculating a score, the remaining values were used for calculating the average score. If more than one value was missing, no score was outputted for that participant.

The MARS questionnaire (participants' evaluation of the impact of the game) was conducted twice: after the intervention, and once again two months later. For each participant, an overall MARS score was calculated by averaging both responses. In case of missing data in one or the other questionnaire (two patients died before the iteration of the questionnaire, and one patient did not answer 2 items the first time but did so the second), the remaining response was kept.

Evaluation of the Quality of the Documentation

For each patient and at each time step (at inclusion and at end-of-study) the documentation was extracted by a research nurse who systematically searched 5 to 10' in the 4 most relevant sections in the HUG medical record system. Three types of ACP documentation were collected: patients' values or goals, preferences for end-of-life care planning, and life-sustaining treatment preferences. Retrieved data were anonymised and printed on separate files. Pre- and post-intervention documentation files were mixed and randomly evaluated by 5 authors (ME, CB, AD, PL, CC). Each type of ACP documentation was evaluated on a 1-5 scale ranging between "not at all clear" and "extremely clear" ("clear" meaning "useful to care and treat the patient if he-she has lost his-her decision-making capacity"). The first five evaluations were discussed in group. The following files were evaluated independently by at least one nurse and one physician, compared and discussed in group in case of divergence.

Statistical analysis

Pre-post changes and patient-nurse group comparisons were performed using Jamovi [24]. All tests were evaluated for statistical significance at alpha level .05. Scores were handled as numerical variables and analysed with paired- or unpaired Welch t-test. The condition of application of the tests were systematically assessed and complementary nonparametric tests were conducted when relevant (Mann-Whitney-Wilcoxon). Coherent results were obtained in all cases; therefore, only t-test results are reported in the result section. For observing within-group changes in frequencies, the contingency McNemar Chi-Square test was used.

To check (as announced in the pre-registration of the study) whether further possible explanatory variables could have impacted on the main results, exploratory X^2 and correlation tests were conducted with the following variables: decision to retract (yes/no), uploaded advance directives (AD) at the end of study (yes/no), changes in pre-post scores on the ACPES scale (1-5), age (nb of years), gender (M/F/Other), education level (Professional apprenticeship/High school and vocational

school/University level), Charlson comorbidity score (nb), hope of being transplanted (yes/no), patient working activity (yes/no), patient supported by a relative (yes/no), French or non-native French language (yes/no). No relevant results came out of these analyses.

Results

Compliance to study procedures

In response to one item of the questionnaire, nurses report having complied to the study procedure (mean=3.6±.84, n=10). However, minor deviations from the protocol were observed and overall ACP counselling and completion of questionnaires took twice more time than expected per study protocol.

Deviations from research protocol were the following: sometimes, nurses failed to inform the research assistant that the ACP procedure was over, which induced a time lag to complete the post-intervention questionnaire. Moreover, on some occasions, nurses left an important time before scheduling the next ACP session. Researchers' notes taken during the project indicate difficulties related to work overload and organizational constraints (patients had to undergo surgery or experienced a temporary worsening of the health conditions or postponed the next meeting). These difficulties are illustrated in the Table below by some aberrant time spent for the ACP intervention (228 days in the maximal case). In other cases, this was due to a lack of compliance on the part of the nurse. Such situations notably happened with nurses that withdrew in the course of the study.

Duration of study phases in number of days

	N	Mean	Median	SD	Min	Max
Duration of the 3-step ACP counselling	19	84	64	66	10	228
Duration between second and end questionnaires	13	89.3	86	33.7	53	166

Planned study phases tended to take more time than expected. According to the study protocol, expected duration of ACP counselling = ±40 days and expected duration between second and end questionnaires = ±60 days. However, counselling time took on average twice more time. Moreover, aberrant durations between the first and second post-intervention questionnaires were observed. Some of these cases are due to technical and organizational reasons, in some cases, patients' answers were first reported on a printed formula and later entered in Redcap. This procedure artificially increased the time lag.

DOI: 10.1016/j.ekir.2025.11.001

Limitations

Our results are based on a small dataset, which could be strongly impacted by chance or other confounding factors.

Per protocol, we did not interview or collect post-intervention questionnaire data from patients that withdrew before having completed ACP consultations. Our dataset is therefore biased towards positive feedback and we obtained little information about barriers or reasons for refusing ACP.

Most of the recruitment was made PL who already knew the nurses and the patients. Some participants may thus have accepted to be included in the study because of personal ties rather than intrinsic motivation. Moreover, the training was done by CB and PL who know the nurses. The research assistant who collected questionnaire answers and retrieved data from medical records, however, was external and thus less likely impacted by researchers' biases.

In some cases, technical and organizational difficulties artificially increased the reported time lag between the first and second post-intervention questionnaires. Therefore, data reporting time between intervention phases are not fully reliable.

Part of qualitative data collection are researchers' note taking during the intervention. These data are vulnerable to researchers' biases and their personal evaluation of what is relevant to take note of. These notes, however, are mostly confirmed by the results of the focus groups and interviews that were designed more rigorously and analysed separately (submitted in a separate paper).
